# Technical feasibility of newborn screening for spinal muscular atrophy by next-generation DNA sequencing

**DOI:** 10.3389/fgene.2023.1095600

**Published:** 2023-01-12

**Authors:** Bennett O. V. Shum, Ilya Henner, Anita Cairns, Carel Pretorius, Urs Wilgen, Paulette Barahona, Jacobus P. J. Ungerer, Glenn Bennett

**Affiliations:** ^1^ Preventive Health Division, Genepath, Sydney, NSW, Australia; ^2^ EMBL Australia Node in Single Molecule Science, School of Biomedical Sciences, University of NSW, Sydney, NSW, Australia; ^3^ Neurosciences Department, Queensland Children’s Hospital, Brisbane, QLD, Australia; ^4^ Department of Chemical Pathology, Pathology Queensland, Queensland Health, Brisbane, QLD, Australia; ^5^ Faculty of Health and Behavioural Sciences, University of QLD, Brisbane, QLD, Australia

**Keywords:** next-generation DNA sequencing, newborn screening, spinal muscular atrophy, SMA, SMN1, analytical validation, genomics

## Abstract

Newborn screening (NBS) assays for spinal muscular atrophy (SMA) typically use a polymerase chain reaction (PCR) based assay to identify individuals with homozygous deletion in exon 7 of the *SMN1* gene. Due to high DNA sequence homology between *SMN1* and *SMN2*, it has previously been difficult to accurately bioinformatically map short reads from next-generation DNA sequencing (NGS) to *SMN1*, resulting in low analytical performance and preventing NGS being used for SMA screening. Advances in bioinformatics have allowed NGS to be used in diagnostic settings, but to date these assays have not reached the scale required for high volume population newborn screening and have not been performed on the dried blood spot samples that NBS programs currently use. Here we integrate an NGS assay using hybridisation-based capture with a customised bioinformatics algorithm and purpose designed high throughput reporting software into an existing NBS program to achieve a laboratory workflow for population SMA screening. We tested the NGS assay on over 2500 newborns born over 2 weeks in a NBS program in a technical feasibility study and show high sensitivity and specificity. Our results suggest NGS may be an alternate method for SMA screening by NBS programs, providing a multiplex testing platform on which potentially hundreds of inherited conditions could be simultaneously tested.

## Introduction

Spinal muscular atrophy (SMA) is an autosomal recessive disorder that causes alpha motor neuron degeneration that leads to progressive muscular weakness and sometimes death and has an incidence of approximately 1 in 10,000 live births (Verhaart et al., 2017). Affected individuals inherit a dysfunctional *SMN1* allele from each biological parent, and the exon 7 deletion is the most common pathogenic variant. The severity of disease is modified by the number of copies (dosage) of the *SMN2* gene, a paralog of *SMN1*, by production of small amounts of full-length transcripts which result in functional protein and a milder phenotype. New and emerging therapeutic options can ameliorate disease progression by increasing *SMN2* gene expression or by replacing *SMN1* function, although clinical benefits are dependent on factors including disease duration (Mercuri et al., 2020). Early diagnosis and treatment of SMA by newborn screening (NBS) is one way to improve clinical outcomes for affected individuals and SMA is routinely screened, or being considered, in countries worldwide (Dangouloff et al., 2021). Two methods for SMA NBS use dried blood spot (DBS) samples in a Real-Time PCR (RT-PCR) or digital droplet PCR (ddPCR) assay to detect a homozygous *SMN1* exon 7 deletion which is responsible for >95% of SMA cases (Taylor et al., 2015; Vidal-Folch et al., 2018; Jiang et al., 2020; Romanelli Tavares et al., 2021). Here we assessed the technical feasibility of using next-generation DNA sequencing (NGS) as an alternate method for NBS programs to screen for SMA.

## Methods

### Study design

As a first step in validating the assay we used a technical feasibility methodology to examine if NGS could accurately discriminate between positive and negative control samples in a blinded group of 12 positive controls and 4 negative controls. Our approach is similar to previous studies that validated PCR-based SMA screening assays, and we used a comparable number of positive controls (Dobrowolski et al., 2012; Taylor et al., 2015; Chien et al., 2017; Vidal-Folch et al., 2018). We then applied our NGS SMA screening test in a whole of population sample of 2552 newborns to determine its suitability for high throughput screening, and to further examine the test’s specificity. A clinical validation study, which was beyond the scope of this study, would need to involve over 120,000 NBS samples under real world conditions to identify the number of positive controls used in the first step of our validation and was not feasible at this stage of the assay’s development.

### Patients and samples

DBS samples were collected from 2552 newborns over a 2-week period in March 2021 from neonates born in 80 maternity units in a population NBS program across the state of Queensland, Australia. Twelve positive and 4 negative control DBS samples were also included in the analysis. Positive controls were deidentified clinical samples from children diagnosed with SMA who had MLPA diagnostic testing at the Queensland Children’s Hospital, Brisbane. The study complied with the Helsinki declaration and the research protocol was approved by the Royal Brisbane and Women’s Hospital, Queensland ethics committee (HREC/2020/QRBW/64125: 03/2021).

### Next-generation DNA sequencing

Genomic DNA was extracted from DBS samples using a Chemagic 360 instrument (PerkinElmer), enzymatically fragmented and amplified by PCR with indexed (barcoded) primers. Pre-capture DNA libraries were hybridized to custom designed SureSelect XTHS2 (Agilent) capture probes to enrich for coding and selected non-coding regions of 176 genes including *SMN1* and *SMN2*. Batched samples from the same capture pool were grouped and processed together. The hybridization-based target enrichment was automated to process >200 DBS per day resulting in a throughput of 1536 samples per week for next-generation DNA sequencing on a Novaseq 6000 (Illumina) system using 300 cycles.

### Primary, secondary, and tertiary DNA analysis

Image analysis was performed in real time by the NovaSeq Control Software v1.7.5 and Real Time Analysis (RTA) v3.4.4. RTA archives primary analysis through real-time base calling on the NovaSeq instrument computer. Illumina DRAGEN BCL Convert 07.021.624.3.10.8 pipeline was used to generate the sequence data. FASTQ sequencing files were aligned to the hg38 human reference genome by ATLAS software (v.0.1.0, Genepath). Sequencing reads were assigned to either *SMN1* or *SMN2* based on 3 genomic positions (*SMN1* chr5:70247724, chr5:70247773, and chr5:70247921) which are unique between the two genes, and exon 7 copy number calculated based on previously published bioinformatics algorithms (Feng et al., 2017; Chen et al., 2020) to allow exon 7 copy number quantification and subsequent identification of the exon 7 deletion for secondary analysis. Tertiary analysis was done on a batch of 1536 samples per week by ATLAS software (v.0.1.0, Genepath) using a semi-automated process based on ACMG DNA variant reporting guidelines, generating a clinical report for samples homozygous for the *SMN1* exon 7 deletion. The software was not designed to detect SMA carriers.

A sample failure occurred if there was insufficient pre-capture library produced or if average read depth coverage of control genes were <20X per sample.

### Sensitivity and specificity analysis

To investigate specificity [=True negative/(True negative + False positive)], we screened a whole of population sample of 2552 children born between 29/03/21 and 13/04/21 none of whom were clinically diagnosed with SMA type 1 or type 2 between 29/03/21 and 10/11/22 using conventional clinical diagnostic pathways at the Queensland Children’s Hospital, which provides the only centralised service for diagnosis of SMA in the state of Queensland. In this technical feasibility study, it was not practical to test 2552 individuals who screened negative with a second assay. Clinical case finding is an accepted (Kariyawasam et al., 2020), although not perfect, method for determining true negatives in newborn screening studies because there are well developed pathways for diagnosing rare diseases in many countries, if a newborn tests negative with a screening assay but develops the disease later in life and has therefore actually had a false negative screen.

To calculate sensitivity [=True positive/True positive + False negative)] of our assay we examined its ability to identify the *SMN1* homozygous exon 7 deletion in a blinded analysis of 12 positive samples from deidentified local samples which had been tested with MLPA (MRC-Holland), which is approximately twice the number of SMA cases diagnosed in the state of Queensland annually, plus 4 blinded negative samples. Because the output of the bioinformatic analysis is qualitative (i.e., homozygous *SMN1* exon 7 deletion present or *SMN1* exon 7 present) confidence intervals for sensitivity and specificity were not applicable.

## Results

**FIGURE 1:**
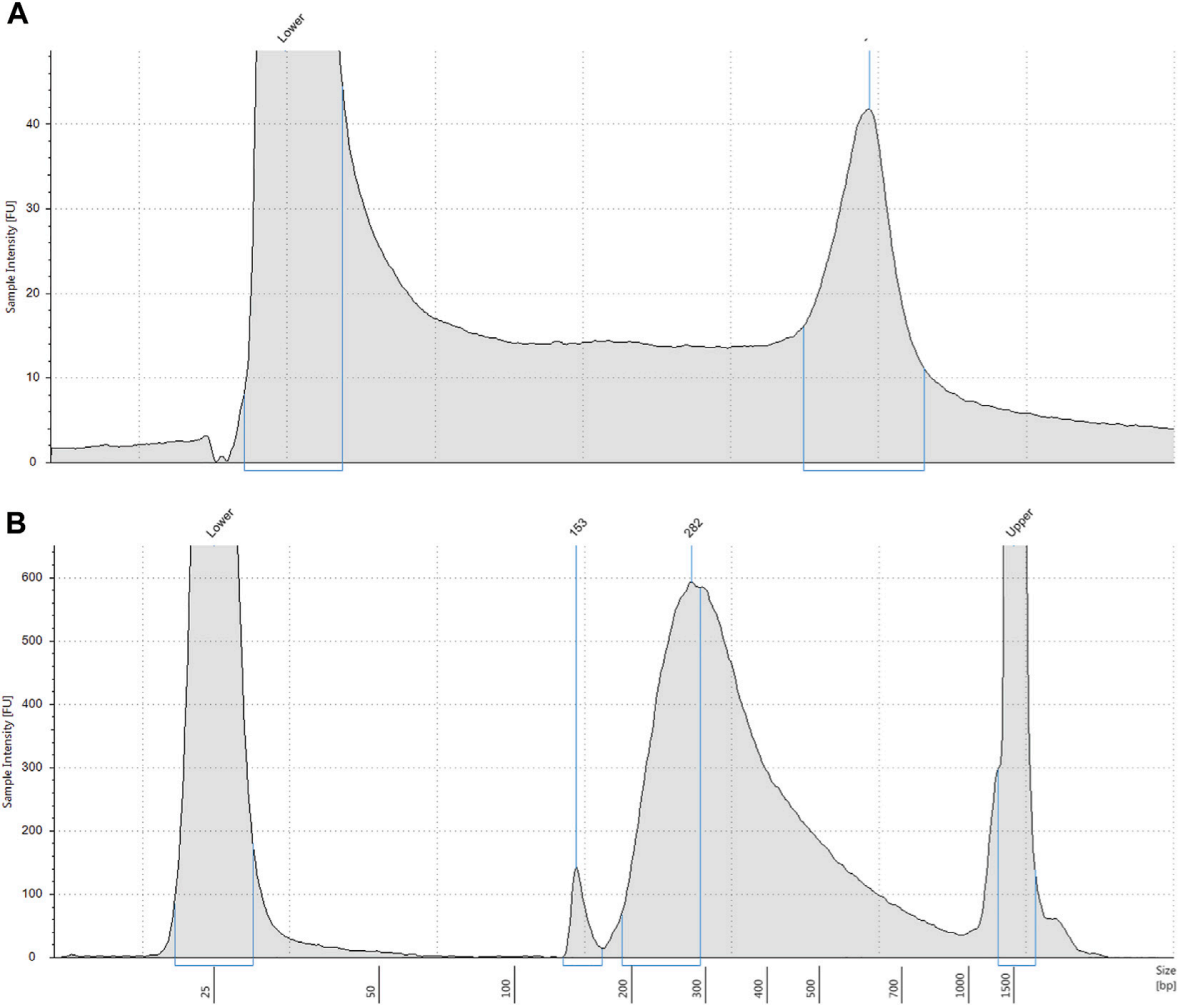
**(A)** Representative electropherogram profile of genomic DNA extracted from DBS. Extracted DNA was analysed on a Tapestation 4200. DNA molecular weight is shown on the *x*-axis and intact high molecular weight DNA is shown by the right peak. Lower = low molecular weight standard. **(B)** Representative electropherogram profile of pre-capture DNA library. DNA library indexed with molecular barcodes was analysed by Tapestation 4200 with a D1000 assay. Most DNA fragments are approximately 282 base pairs. Lower = low molecular weight standard. Upper = high molecular weight standard.

We obtained an average of 3 ng/μL (SD 0.9) of genomic DNA from the control samples, and an average of 5.2 ng/μL (SD 3.2) of pre-capture DNA library after library preparation (Figures 1A, B). Sequencing yielded an average of 469,518 paired end reads (SD 200,480) per sample which equated to an average read depth coverage of 179X (SD 80) for control genes across the 16 samples, and uniformity of coverage for each sample was >95%. Twelve samples were correctly identified to be positive for the homozygous deletion (*n* = 24 alleles) and the remaining 4 samples were classified as either a carrier only or did not harbour the exon 7 deletion. We found the sensitivity of the assay was 100% (Table 1).

**TABLE 1:** Results of SMA NGS screening of blinded positive and negative controls.

Sample type	Screen positive (homozygous exon 7 deletion)	Screen negative (either carrier or no exon 7 deletion)	Assay result
Confirmed SMA	True positive = 12	False negative = 0	Sensitivity = 100%
No SMA	False positive = 0	True negative = 4	Specificity = 100%

**FIGURE 2:**
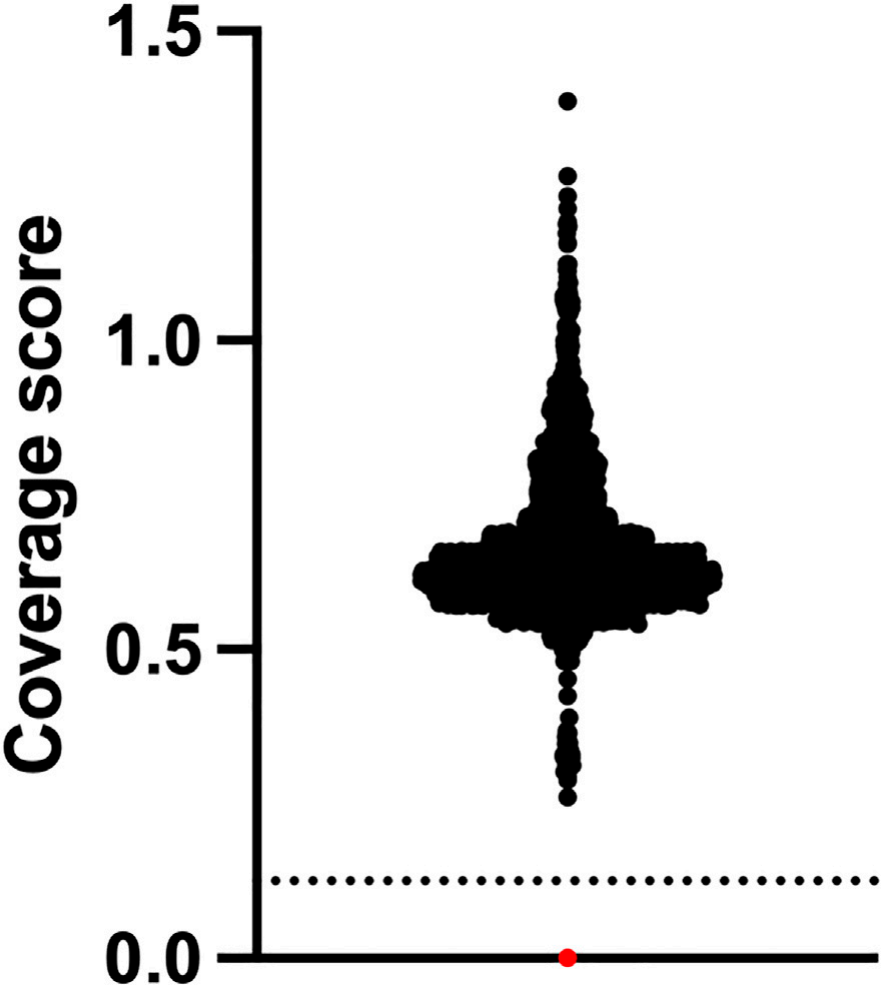
SMA newborn screening using NGS has 100% specificity. Newborns (*n* = 2,552) who were clinically unaffected by SMA at approximately 18 months of age underwent NGS of the *SMN1* gene and relative copy number analysis of exon 7 which is expressed as a coverage score per sample. Positive samples have a homozygous exon 7 deletion and are found below the dotted line cut-off value. Black dots are negative samples. The red dot is a Coriell SMA positive control sample NA09677.

The assay did not identify any positive cases from the 2552 whole of population samples screened, which equates to a specificity of 100%. One positive control sample NA09677 was clearly identified as harbouring the homozygous exon 7 deletion by our NGS assay (Figure 2). From a total of 2568 samples used for this study, the failure rate was 0%. The cost of reagents and software for testing was approximately USD80 per sample. Setup capital and labour costs are not included.

## Discussion

Our results show NGS is a highly sensitive and specific method for primary SMA screening, with 100% sensitivity and specificity in detecting the homozygous *SMN1* exon 7 deletion in over 2500 samples with a sample failure rate of 0%. We developed laboratory and bioinformatics software automation procedures that can screen over 200 samples a day using NGS, to generate a weekly batch size of 1536 DBS samples.

Whilst there has been recent interest in using whole genome NGS in reproductive carrier screening for SMA and for diagnostic testing in a low throughput setting (Chen et al., 2020), the high sensitivity and specificity found in our study supports technical feasibility of implementing targeted genomic NGS for the application of newborn screening for SMA where high throughput laboratory workflows, data analysis, and clinical reporting are all required.

Screening for SMA using RT-PCR is one method used by NBS programs, but this approach may lead to high rates of false positive results in certain assays (Chien et al., 2017), whereas we had no false positive results after screening 2552 samples. One reason for this may be that current RT-PCR assays use only 1 locus to discriminate between *SMN1* and *SMN2* sequences. We found NGS to have 100% specificity for SMA screening when 3 loci are used to assign a sequencing to read to *SMN1* or *SMN2*. Our feasibility study shows NGS to be a robust method for SMA screening, with all samples meeting quality metrics for DNA library preparation and sequencing and no re-testing of any sample was required.

The NGS assay we developed screens for the exon 7 deletion which is responsible for >95% of SMA cases. Current PCR-based assays also only screen for this variant. Future studies examining if NGS can also identify disease-causing SNVs in *SMN1* are warranted to determine whether the clinical sensitivity of the NGS assay may be improved beyond that of PCR-based assays.

Hybrid genes and rearrangements may occur in *SMN1* and potentially cause incorrect results from both PCR and NGS assays. In Australia all children have confirmatory diagnostic testing with MLPA after a positive screening test to identify false positive screen results.

Furthermore, the assay was designed for first tier screening of SMA and not for diagnostic or prognostic purposes and *SMN2* copy number calculation was beyond the scope of this validation study. Studies investigating if NGS can simultaneously output *SMN2* copy number in SMA positive screens may be useful to clinicians because *SMN2* copy number is used to guide early treatment of SMA, thereby improving the clinical utility of NGS assays for SMA screening.

One challenge with expanding NBS is the cost of implementing single disease assays into existing programs. NGS allows multiplex testing for potentially hundreds of conditions which improves the cost effectiveness of testing. Other conditions currently screened using biochemical assays or other conditions that meet Jungner and Wilson criteria for screening for which there is currently no screening test available warrant investigation as candidate conditions for inclusion in an NGS assay.

In the current study the cost for the entire panel of 176 genes was less than USD80 per individual, or less than USD1 per condition screened. The cost for existing PCR-based testing for a single condition, SMA, is approximately USD5 per individual (Romanelli Tavares et al., 2021; Velikanova et al., 2022). The overall cost for implementing NGS testing for SMA into current NBS programs would therefore be greater than that to implement PCR-based testing, but we envisage NGS may be more cost effective overall because of the ability to screen for multiple conditions in one assay.

In summary, we show that NGS may be an alternative technology to RT-PCR or ddPCR for NBS programs to consider when implementing SMA screening.

## Data Availability

The datasets presented in this study can be found in online repositories. The names of the repository/repositories and accession number(s) can be found below: NCBI, BioProject ID PRJNA904501.
